# Calixazulenes: azulene-based calixarene analogues – an overview and recent supramolecular complexation studies

**DOI:** 10.3762/bjoc.14.225

**Published:** 2018-09-25

**Authors:** Paris E Georghiou, Shofiur Rahman, Abdullah Alodhayb, Hidetaka Nishimura, Jaehyun Lee, Atsushi Wakamiya, Lawrence T Scott

**Affiliations:** 1 Department of Chemistry, Memorial University of Newfoundland, St. John’s, Newfoundland and Labrador A1B 3X7, Canada; 2 Aramco Laboratory for Applied Sensing Research, King Abdullah Institute for Nanotechnology, King Saud University, Riyadh, Saudi Arabia; 3 Department of Physics and Astronomy, College of Science, King Saud University, Riyadh 11451, Saudi Arabia; 4 Institute for Chemical Research, Kyoto University, Uji, Japan; 5 Merkert Chemistry Center, Boston College, Chestnut Hill, MA, 02467 USA

**Keywords:** azulene, calixarenes, calixazulenes, supramolecular chemistry, tetraalkylammonium salts

## Abstract

Some of the least studied calixarenes are those that consist of azulene rings bridged by -CH_2_- groups. Since Lash and Colby’s discovery of a simple and convenient method for producing the parent all-hydrocarbon calix[4]azulene, there have been two other all-hydrocarbon calix[4]azulenes which have been synthesized in good yields by their method. This allowed studying their supramolecular properties. This report is of our latest work on the solution-state supramolecular complexation of one of these calix[4]azulenes, namely tetrakis(5,7-diphenyl)calix[4]azulene or “OPC4A”, with several electron-deficient tetraalkyammonium salts. As a result of more recent methods developed by us and others employing Suzuki–Miyaura cross-coupling reactions to produce additional functionalized azulenes, the promise of further greater functionalized calixazulenes lies in store to be investigated.

## Introduction

Among the great variety of synthetic macrocyclic molecular receptors which have been reported, those that are referred to by their generic name “calixarene” loom large [1–3]. The relatively facile and reproducible syntheses of the classical calix[*n*]arenes **1** in which *n* = 4, 6 or 8, with phenolic groups linked or bridged via methylene groups to form defined three-dimensional basket-like cavities with “upper” or “lower” rims, were developed by Gutsche and co-workers [4–6]. As a result of Gutsche’s synthetic methodologies many researchers have been able to employ these calix[*n*]arenes and modified derivatives thereof in a great variety of ingenious applications. These applications have included a myriad of synthetic modifications to both, or either, of their upper and lower rims, and also to their bridging methylene groups, all of which have resulted in further synthetic endeavours. Much of the groundwork for these endeavours have resulted from the pioneering works which emanated from the research groups of C. D. Gutsche, R. Ungaro, D. N. Reinhoudt, and V. Böhmer to name only just a few. Reinhoudt has recently presented an overview of the historical evolution of the chemistry of the calixarenes [1]. Supramolecular applications, in particular, of many of the great number of creative derivatives of calixarenes which have been and continue to be synthesized are widely being reported in the literature [7].

**Figure 1:**
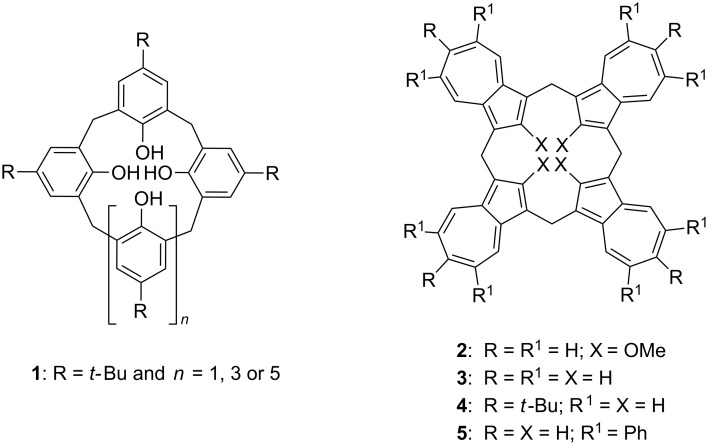
Examples of calix[*n*]arenes **1** and calix[4]azulenes **2**–**5**.

Besides the classical calixarene phenolic subunits linked by methylene groups, “calixarenes” incorporating other subunits include, but are not limited to, resorcinol [8], hydroquinone [9], naphthols [10], pyrrole [11], heteroaromatics [12] and triptycene [13] in their cavity-containing structures have gained much recent attention. Among the least-studied to date, however, have been the azulene unit-containing calix[4]arene analogues. In 1988 Asao et al. reported the synthesis of the first azulene analogue of the calixarenes, which they called “azulenophane” **2** [14]. They used a semi-convergent route and reported that **2** had a *1,3-alternate* conformation at room temperature and that it “formed crystals with two molecules of benzene” but they reported no other studies. To the best of our knowledge, this is the only “lower-rim” functionalized calix[4]azulene which has been reported to date. In 2002 Lash and Colby’s reported a convenient one-step Florisil^®^-mediated cyclocondensation of azulene with paraformaldehyde to produce an all-hydrocarbon “calix[4]azulene” **3** [15]. Later, Lash et al. reported their synthesis of a second all-hydrocarbon tetra-6-*tert*-butylcalix[4]azulene (**4**) in a similar way, from the reaction of 6-*tert*-butylazulene with formaldehyde [16]. Compound **4** is the first reported “wide-rim” functionalized calix[4]azulene (Figure 1).

Recently, we reported the synthesis of tetrakis(5,7-diphenyl)calix[4]azulene (**5**) (or octaphenylcalix[4]azulene, “OPC4A”**,** Figure 1) and on its mechanochemically-generated solid-state complex of C_60_-fullerene [17]. This all-hydrocarbon, wide-rim octaphenyl-functionalized calix[4]azulene was designed to evaluate its potential for encapsulating C_60_ or C_70_ fullerenes. The lack of sufficient solubility of **5** in common organic solvents prevented a fuller examination of its potential supramolecular properties with fullerenes, a topic of particular interest to us [18]. Therefore, the solid state supramolecular complexation properties of **5** were experimentally studied using solid state NMR and XRD experiments, and also theoretically, using a DFT analysis [17]. We previously used a similar solid-state NMR approach to study the solid-state supramolecular properties of tetra-6-*tert*-butylcalix[4]azulene (**4**) [19]. Unlike these two studies, however, in our first study on calixazulenes which we reported in 2015, we were able to demonstrate a chloroform solution-state complexation binding study with Lash and Colby’s calix[4]azulene **3** using a series of tetraalkylammonium halides and tetrafluoroborate salts [20]. This study was also supplemented by DFT studies to support the trends observed in the experimentally-derived binding constants. Since these three calix[4]azulenes **3**–**5** are all-hydrocarbon compounds they differ significantly from the better-studied calix[4]arenes, which usually have some heteroatoms such as oxygen, nitrogen or sulfur in their structures. As a consequence, compounds **3**–**5** have solubility limitations. Furthermore, the absence of heteroatoms, most commonly hydroxy groups on the “lower” or narrow rim, also limits their “pre-organizational” potential for supramolecular binding, this being of particular interest to us. We now report that we have succeeded in extracting binding constant data from a solution-state UV–vis supramolecular binding study recently concluded with OPC4A. These results and a corresponding DFT study are reported herein.

## Results and Discussion

The convenient synthesis of the precursor for OPC4A **5**, namely 5,7-diphenylazulene, which is normally a difficult target molecule, was previously described from a Suzuki–Miyaura coupling reaction of bromobenzene with 5,7-di(Bpin)azulene, which in turn was formed via the exhaustive borylation of azulene with excess bis(pinacolato)diboron (B_2_pin_2_) [21]. Cyclocondensation of 5,7-diphenylazulene with formaldehyde produced **5** [22] under conditions similar to those used by Lash and Colby in their syntheses of **3** and **4**. Although **5** was not sufficiently soluble in CS_2_, benzene, toluene or 1,2-dichlorobenzene to enable ^1^H NMR solution titration studies to be conducted with fullerene C_60_, a dilute solution of **5** in dichloromethane-*d*_2_ could be obtained that enabled its NMR characterization. This finding suggested to us that solution complexation studies with other electron-deficient suitable guests could be conducted in dichloromethane (DCM). The concentrations that could be obtained with DCM were too dilute for typical NMR titration studies, but we judged that they could instead be suitable for a UV–vis titration study**.** Indeed, after several preliminary trials, solutions of approximately 1.2 mg of **5** in 100.0 mL of DCM (≈ 1.1 × 10^−5^ M) could eventually be generated with the help of sonication in a 35 °C water-bath. By way of contrast, initial attempts to create more concentrated solutions in chloroform under similar and higher temperature (60 °C) sonication conditions resulted in the unexpected decomposition of **5**, a finding which was not investigated any further.

With DCM solutions of OPC4A now in hand, titration studies were conducted using 1.0 cm pathlength cells in a thermostated dual beam UV–vis spectrophotometer. Addition of microlitre aliquots of DCM solutions of the respective tetraalkylammonium salts (TRAX; where R = Me, Et; *n*-Bu and X = Cl^−^, Br^−^, I^−^ or BF_4_^−^) resulted in quenching of the absorption spectra in the 300–700 nm range, with visible isosbestic points at ≈460 and 350 nm. Although the changes were small, as was also seen previously in the titration experiments with **3**, they were sufficient to allow for reproducible determinations of the corresponding apparent *K*_assoc_ values. Each of the full spectra could be subjected to non-linear 1:1 global fit analyses as described by Thordarson [23–24].

Table 1 shows the measured apparent binding or association constants, from which two trends can be discerned: Firstly, the *K*_assoc_ values with the tetra-*n*-butylammonium halide salts show a trend that is in the order Cl^−^ > Br^−^ > I^−^. This trend is similar to that seen previously with the corresponding tetramethylammonium halides and **3**. Secondly, with respect to the tetraalkylammonium BF_4_ salts, the corresponding *K*_assoc_ trend is in the order *n*-Bu > Et > Me. This trend is in contrast and opposite to that which was seen previously with the unfunctionalized calix[4]azulene **3**.

**Table 1:** Apparent experimentally-derived binding constants and DFT-computed interaction energies (*IE*) and selected interatomic distances derived from the geometry-optimized structures of the supramolecular complexes and their constituents.^a^

	*K*_assoc_ ± 15% (M^−1^)	*IE* (kJ mol^−1^)	avg. N···C^*^ dist. in complex (Å)	X···N dist. free guest (Å)	X···N dist. in complex (Å)	Δ X···N dist. (Å)

TBACl	4.4 × 10^4^	−337.805^b^	7.14 ± 0.68	3.79	3.89	0.095
TBABr	3.8 × 10^4^	−315.073^b^	7.13 ± 0.67	4.07	4.14	0.081
TBAI	2.9 × 10^4^	−316.402^b^	7.06 ± 0.66	4.47	4.34	0.13
TMABF_4_	4.8 × 10^3^	−155.935^c^	4.78 ± 0.18	3.98	4.13	0.15
TEABF_4_	3.3 × 10^4^	−164.812^c^	5.76 ± 0.45	3.96	4.11	0.15
TBABF_4_	4.1 × 10^4^	−198.832^c^	7.09 ± 0.68	3.97	4.10	0.13

^a^TBAX: tetra-*n*-butylammonium halide where X = Cl, Br or I; TRABF_4_: tetraalkylammonium fluoroborate where R = M = methyl; R = E = ethyl or R = B = *n*-butyl. ^b^Value derived using ωB97xD/GenECP and ^c^Value derived using ωB97xD/6-31G(d).

To shed light on possible explanations for these findings, our attention was again directed to computational results derived from DFT calculations which are increasingly being commonly used in supramolecular chemistry. The ωB97xD functional [25] which combines the long range functional ωB97x with the empirical dispersion correction was used with the standard 6-31G(d) basis set [26]. We had previously described the use of this system in our previous studies in particular, in reference [20] as being more reliable than the use of B3LYP/6-31G(d) with our systems. Furthermore, for the halide guests and complexes (i.e., for TBACl, TBABr and TBAI) but not with the tetrafluorborate salts, we used relativistic ECPs by Hay and Wadt (LANL) along with the corresponding LANL2DZ basis set augmented with additional *d-, p*-polarizational functions [27–30]. For the TBABF_4_ salts the ωB97xD/6-31G(d) route was used (see Table 1 and Supporting Information File 1). For each of the individual components, i.e., the tetra-*n*-butylammonium salt, OPC4A and the respective corresponding 1:1 supramolecular complexes, unconstrained geometry optimizations were first conducted in the gas phase. Then, geometries in all cases were optimized within the continuum solvation model (PCM) [31–32] of the DCM solvent, using the default solvent parameters as provided with Gaussian-09 Revision E.01 [33]. The results are summarized in Table 1 and Table 2.

**Table 2:** DFT computed energy values for the three different conformations of **5**.

structure	designation	RωB97XD energy (Hartrees)	relative energies (kJ mol^−1^)

**5c**	*saddle*	−3543.128099	0
**5b**	*cone*	−3543.11020759^a^ and −3543.0596655^b^	46.97^a^ and 42.94^b^
**5a**	*1,2-alternate*	−3543.108789	50.70

^a^Value derived using ωB97xD/6-31G(d) and ^b^value derived using ωB97xD/GenECP.

**Figure 2:**
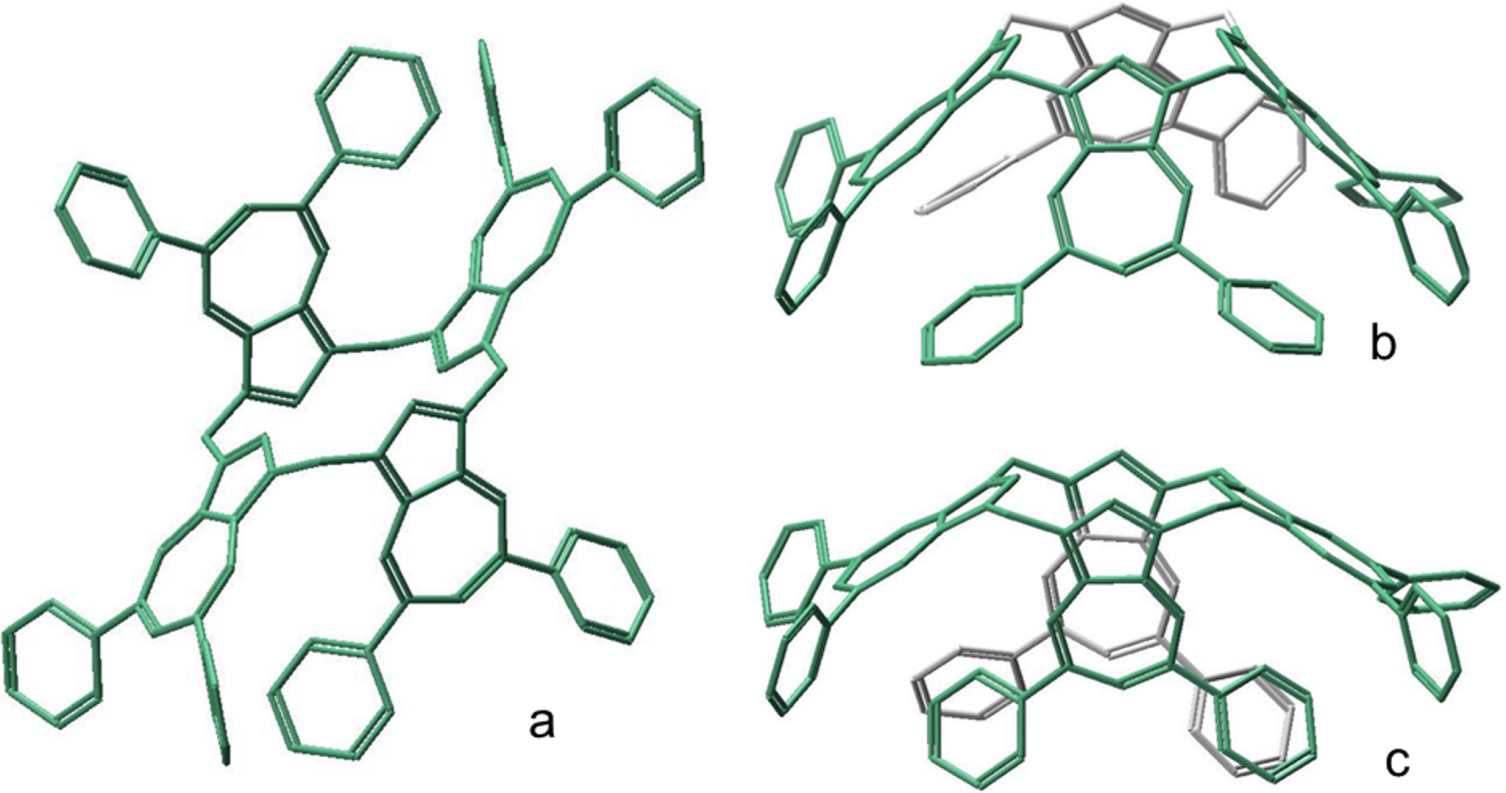
Three major computed conformers of OPC4A; a: *1,2-alternate*; b: *cone* and c: *saddle.*

For the free OPC4A host molecule, initial geometry-optimized determinations were made on the possible major conformations, based upon those previously defined in reference [20]. Three distinct conformations (*saddle*, *cone* and *1,2-alternate*) shown in Figure 2, were generated.

**Figure 3:**
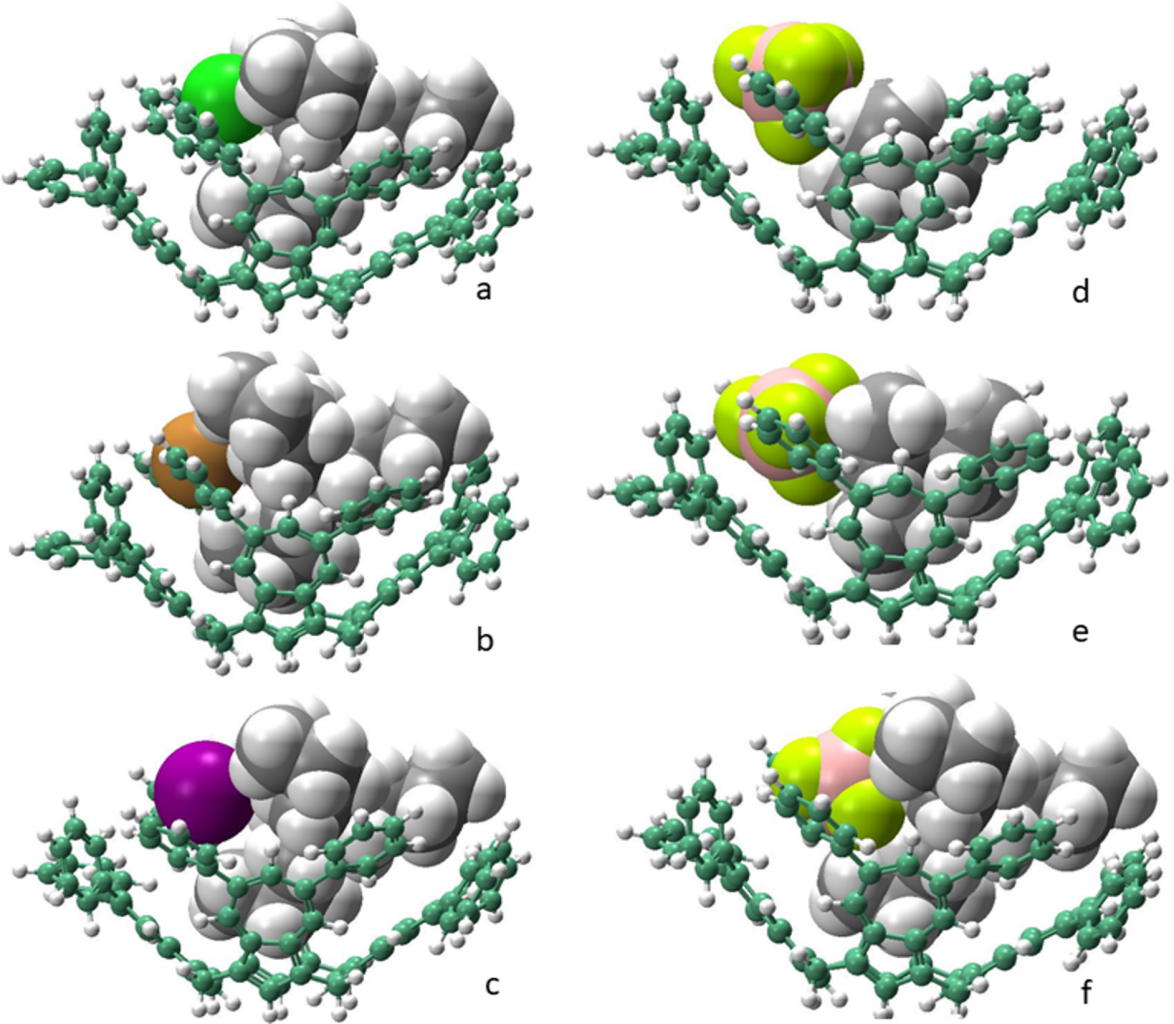
Geometry-optimized (ωB97xD/6-31G(d)) and (ωB97xD/GenECP) structures, respectively, computed for left: (a) **5**
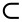
TBACl; (b) **5**
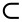
TBABr; and (c) **5**
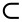
TBAI; right: (d) **5**
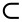
TMABF_4_; (e) **5**
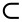
TEABF_4_; and (f) **5**
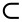
TBABF_4_.

Significantly, whereas for **3** which was based upon its X-ray structure, a *partial cone* conformer could be generated and provided a geometry-optimized energy value, the analogous *partial cone* conformation of **5** could not be similarly geometry-optimized. Instead, for **5**, geometry-optimization produced the *1,2-alternate* form shown in Figure 2a. The energies computed with DCM corrections are shown in Table 2 with the *saddle* conformer (Figure 2c) having the lowest energy. Nevertheless, when subjected to geometry optimizations with the individual respective TRAX salt guests, the saddle conformer opened up to generate and accommodate each of the guests in typical “guest-in-*cone*” structures, as can be seen in Figure 3.

The interaction energies (*IE*) were calculated from the corresponding DFT-calculated geometry-optimised components (i.e., each of **5** and the respective guest TRAX) as 1:1 complexes according to Equation 1:

[1]
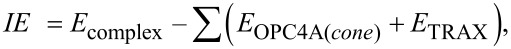


based upon the respective “*cone*” conformation (Figure 2b) energies. These values are shown in Table 1. No easily discernable significant correlation between the interaction energies and the experimentally measured binding constants can be discerned for the three halide salt complexes; the highest *IE* (−337.805 kJ mol^−1^) was found for the chloride which also had the highest binding constant but the corresponding values for the bromide and iodide salts showed no such correlation. The correlations between the *IE*s and binding constants for the tetrafluoroborate salts, however, are more easily discernable and have the same trends in the order of TBABF_4_ > TEABF_4_ > TMABF_4_. The counterion effects of the halide anions are more significant than those of the fluoroborate anion which is weakly coordinating in the salts employed. This can be seen in Table 1 for the relatively smaller changes in the boron-to-nitrogen distances in the DFT-computed optimized geometry structures of the complexes.

Table 1 also shows the average values of the calculated distances between the quaternary nitrogen atom and the “deepest” carbon atoms (i.e., C-1) in each of the azulenes in the calix[4]azulene bowls. A small trend can be discerned for the halide salt complexes which is opposite to the trend in the measured apparent binding constants. For the tetrafluoroborate salts, however, the trend of the corresponding average quaternary nitrogen-to-carbon distances are in the opposite direction, which is consistent with the increasing sizes of the alkyl groups *n*-Bu > Et > Me. Clearly, the BF_4_ salts show less ambiguous DFT data than those of the halide salts in this study. As can be seen in structures d–f in Figure 3, there are more guest C–H_(guest)_–π_(host)_ interactions possible as the size of the alkyl groups increase from groups Me < Et < *n*-Bu, which could also account for the observed trend in their binding constants.

## Conclusion

Based upon the DFT calculations which we previously conducted in the solid-state study of **5** with C_60_, we postulated that due to the mechanochemical method of combining both components and the spherical nature of C_60_ that a possible interaction mode between host and guest could be as columnar arrays [17]. In this type of array the host molecules which are in *1,3-alternate* conformations align in a “head-to-tail” fashion with the C_60_ molecules able to be accommodated within the opposite clefts. Furthermore, within such an arrangement, in addition to the “face-to-face” π–π interactions between the azulene rings and the C_60_, “edge-to-face” type interactions with the 2′,6′-protons of the phenyl group substituents of the azulenes are also factors which could stabilize the solid-state supramolecular interactions or complexation. In the present study, however, due to the dilute solution state conditions, only 1:1 complexation modes between **5** and the respective tetraalkylammonium salts was considered. The binding constants were consistent with such an hypothesis. As a result, the DFT-generated complexes considered only such 1:1 “guest in cone” complexes, as shown in Figure 3a–f. Finally, in light of recent developments in the facile syntheses of other functionalzed azulenes as reported by Narita et al. [34] the potential for further syntheses of hetero-functionalized calixazulenes and their supramolecular chemistry may be realized. Further studies by us on these intriguing possibilities are ongoing.

## Supporting Information

File 1 Experimental determination of binding constants and DFT calculations.

File 2 MOL files.
