# Supplementary material for: Calixazulenes: azulene-based calixarene analogues – an overview and recent supramolecular complexation studies
Source: Beilstein J Org Chem. 2018 Sep 25;14:2488–94. doi: 10.3762/bjoc.14.225 (PMC6178307; doi:10.3762/bjoc.14.225)
Supplement: File 1 — Experimental determination of binding constants and DFT calculations. [file Beilstein_J_Org_Chem-14-2488-s001.pdf]

Supporting Information

for

Calixazulenes: azulene-based calixarene analogues – an overview and recent supramolecular complexation studies

Paris E. Georghiou*[,1], Shofiur Rahman[1,2], Abdullah Alodhayb[2,3], Hidetaka Nishimura[4], Jaehyun Lee[4], Atsushi Wakamiya[4] and Lawrence T. Scott[5]

Address: [1]Department of Chemistry, Memorial University of Newfoundland, St. John's, Newfoundland and Labrador, Canada A1B 3X7, [2]Aramco Laboratory for Applied Sensing Research, King Abdullah Institute for Nanotechnology, King Saud University, Riyadh, Saudi Arabia, [3]Department of Physics and Astronomy, College of Science, King Saud University, Riyadh 11451, Saudi Arabia, [4]Institute for Chemical Research, Kyoto University, Uji, Japan and [5]Merkert Chemistry Center, Boston College, Chestnut Hill, MA, 02467 USA.

Email: Paris E. Georghiou - parisg@mun.ca

*Corresponding author

Experimental determination of binding constants and DFT calculations

Table of contents

	Page
Figure S1: UV–vis spectra of **5** showing 1:1 binding model for **5** with TBACl	S2
Figure S2: UV–vis spectra of **5** showing 1:1 binding model for **5** with TBABr	S3
Figure S3: UV–vis spectra of **5** showing 1:1 binding model for **5** with TBAI	S4
Figure S4: UV–vis spectra of **5** showing 1:1 binding model for **5** with $TMABF_4$	S5
Figure S5: UV–vis spectra of **5** showing 1:1 binding model for **5** with $TEABF_4$	S6
Figure S6: UV–vis spectra of **5** showing 1:1 binding model for **5** with $TBABF_4$	S7
References	S7
Table SI1 DFT calculations	S8

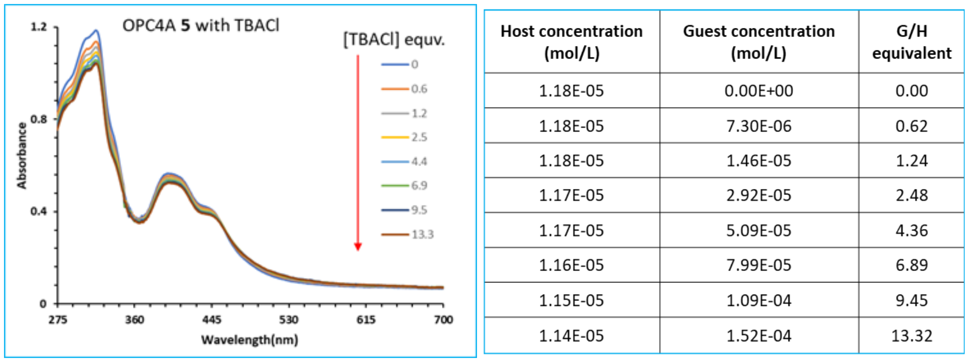

Host concentration (mol/L)	Guest concentration (mol/L)	G/H equivalent
1.18E-05	0.00E+00	0.00
1.18E-05	7.30E-06	0.62
1.18E-05	1.46E-05	1.24
1.17E-05	2.92E-05	2.48
1.17E-05	5.09E-05	4.36
1.16E-05	7.99E-05	6.89
1.15E-05	1.09E-04	9.45
1.14E-05	1.52E-04	13.32

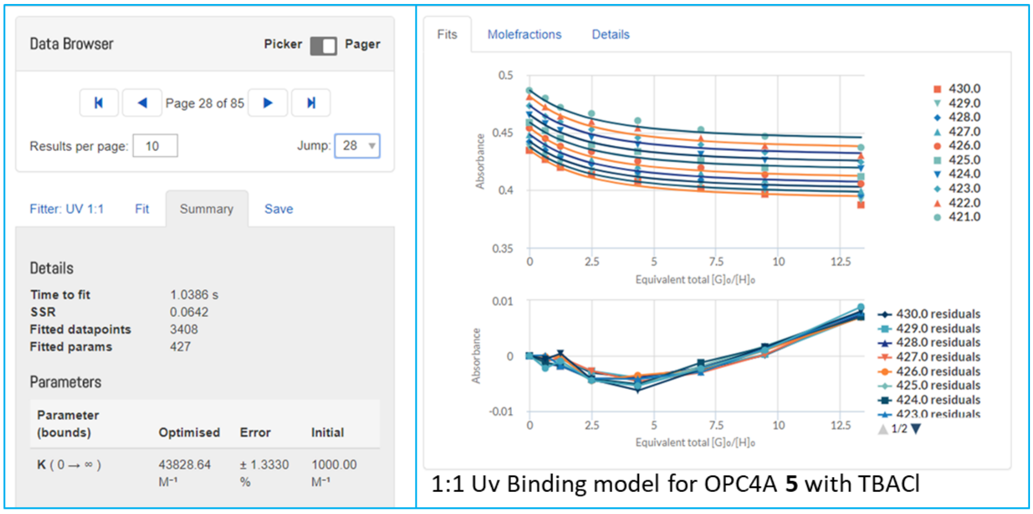

1:1 Uv Binding model for OPC4A **5** with TBACl

Figure S1: *Top: (left and right side)*: UV–vis spectra of OPC4A **5** (1.18×10^{-5} M to 1.14×10^{-5} M) upon addition of TBACl (0–13 equivalents) in 9:1,v/v CH_2Cl_2/CH_3OH. *Bottom:* Screenshot from http://app.supramolecular.org/bindfit/ showing 1:1 binding model for OPC4A **5** with TBACl.

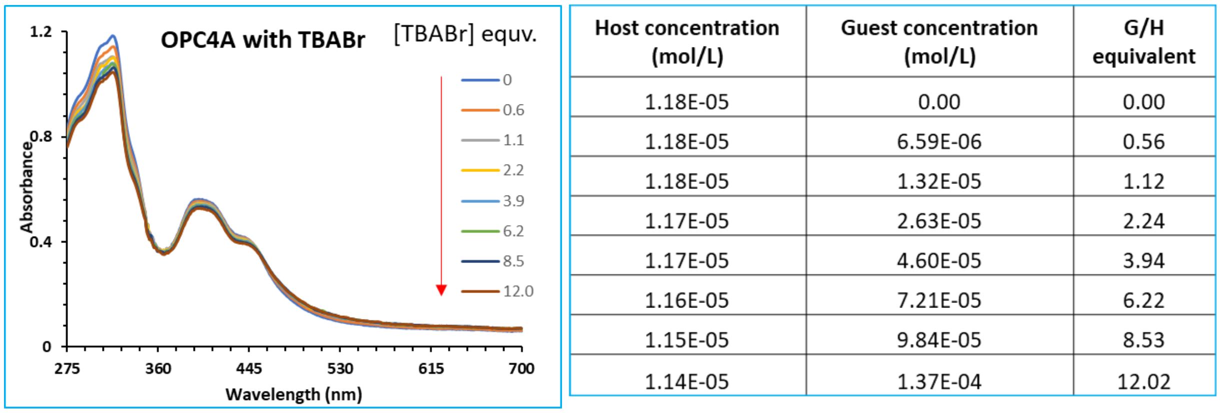

Host concentration (mol/L)	Guest concentration (mol/L)	G/H equivalent
1.18E-05	0.00	0.00
1.18E-05	6.59E-06	0.56
1.18E-05	1.32E-05	1.12
1.17E-05	2.63E-05	2.24
1.17E-05	4.60E-05	3.94
1.16E-05	7.21E-05	6.22
1.15E-05	9.84E-05	8.53
1.14E-05	1.37E-04	12.02

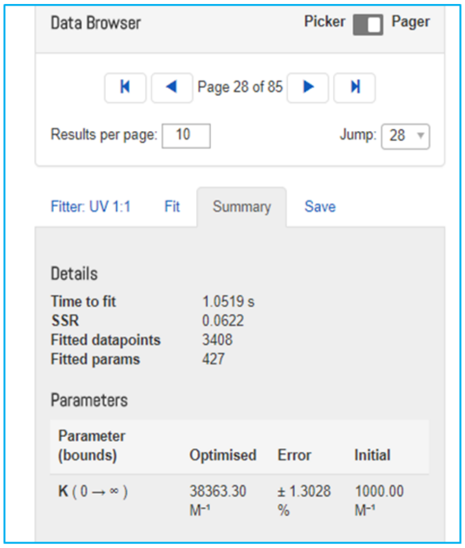

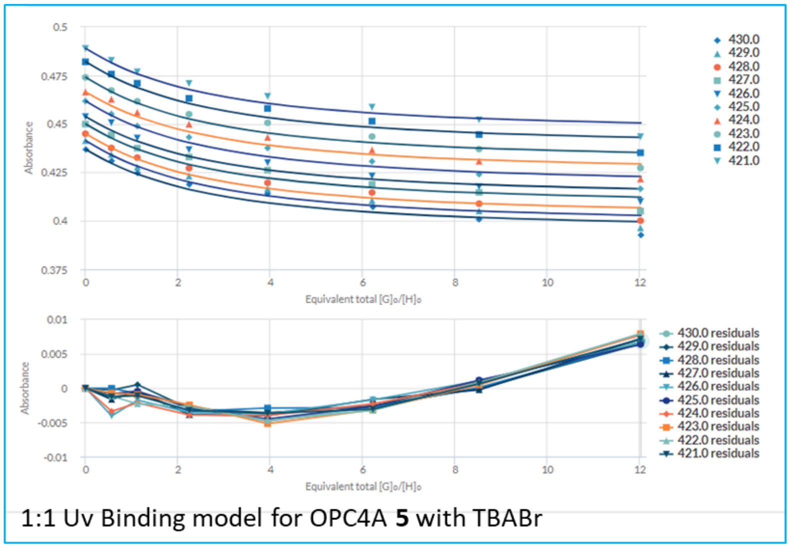

Figure S2: *Top: (left and right side)*: UV–vis spectra of OPC4A **5** (1.18×10^{-5} M to 1.14×10^{-5} M) upon addition of TBABr (0–12 equivalents) in 9:1,v/v CH_2Cl_2/CH_3OH. *Bottom:* Screenshot from http://app.supramolecular.org/bindfit/ showing 1:1 binding model for OPC4A **5** with TBABr.

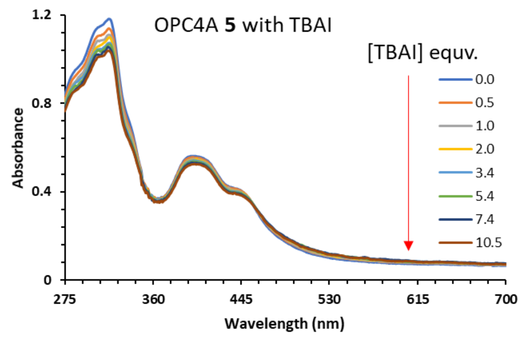

Host concentration (mol/L)	Guest concentration (mol/L)	G/H equivalent
1.18E-05	0.00E+00	0.00
1.18E-05	5.75E-06	0.49
1.18E-05	1.15E-05	0.98
1.17E-05	2.30E-05	1.96
1.17E-05	4.01E-05	3.43
1.16E-05	6.29E-05	5.42
1.15E-05	8.58E-05	7.44
1.14E-05	1.20E-04	10.49

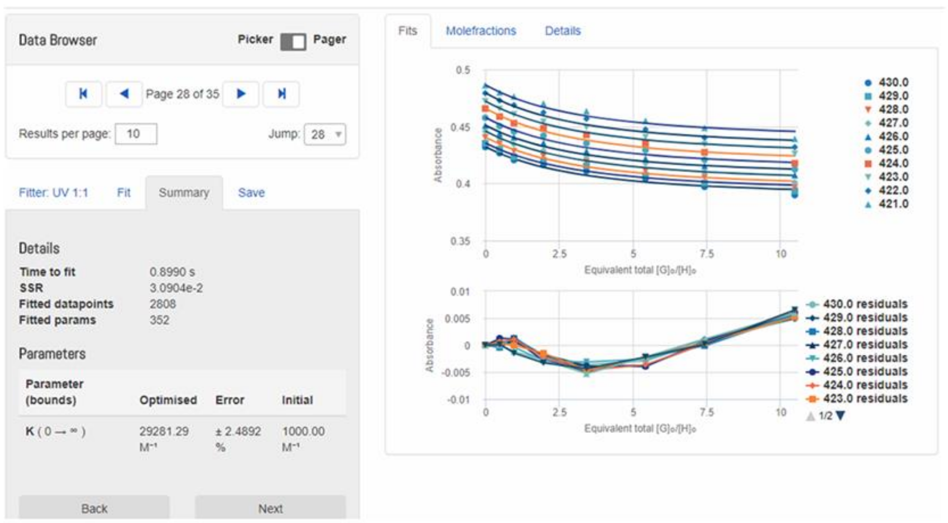

1:1 Uv Binding model for OPC4A **5** with TBAI

Figure S3: *Top: (left and right side)*: UV–vis spectra of OPC4A **5** (1.18×10^{-5} M to 1.14×10^{-5} M) upon addition of TBAI (0–10.4 equivalents) in 9:1,v/v CH_2Cl_2/CH_3OH. *Bottom:* Screenshot from http://app.supramolecular.org/bindfit/ showing 1:1 binding model for OPC4A **5** with TBAI.

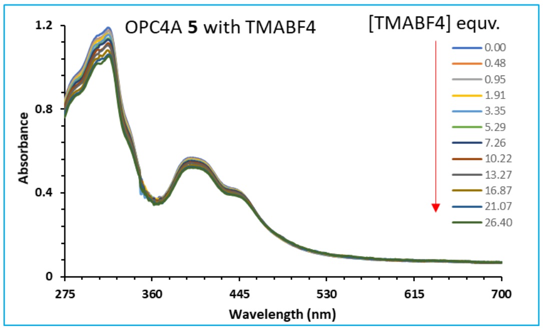

Host concentration (mol/L)	Guest concentration (mol/L)	G/H equivalent
1.18E-05	0.00E+00	0.00
1.18E-05	5.75E-06	0.49
1.18E-05	1.15E-05	0.98
1.17E-05	2.30E-05	1.96
1.17E-05	4.01E-05	3.43
1.16E-05	6.29E-05	5.42
1.15E-05	8.58E-05	7.44
1.14E-05	1.20E-04	10.49

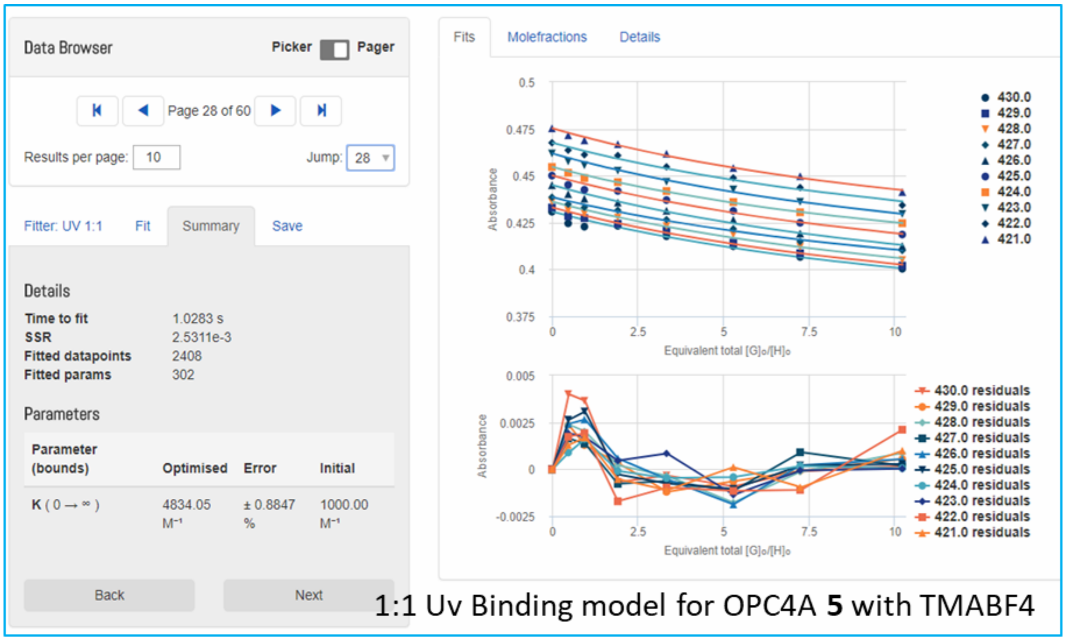

Figure S4: *Top: (left and right side)*: UV–vis spectra of OPC4A **5** (1.18×10^{-5} M to 1.14×10^{-5} M) upon addition of $TMABF_4$ (0–10 equivalents) in 9:1,v/v CH_2Cl_2/CH_3OH. *Bottom:* Screenshot from http://app.supramolecular.org/bindfit/ showing 1:1 binding model for OPC4A **5** with $TMABF_4$

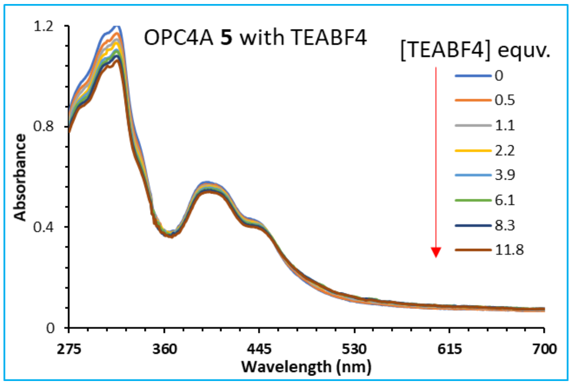

Host concentration (mol/L)	Guest concentration (mol/L)	G/H equivalent
1.18E-05	0.00E+00	0.00
1.18E-05	6.45E-06	0.55
1.18E-05	1.29E-05	1.10
1.17E-05	2.58E-05	2.19
1.17E-05	4.50E-05	3.85
1.16E-05	7.06E-05	6.08
1.15E-05	9.62E-05	8.35
1.14E-05	1.34E-04	11.76

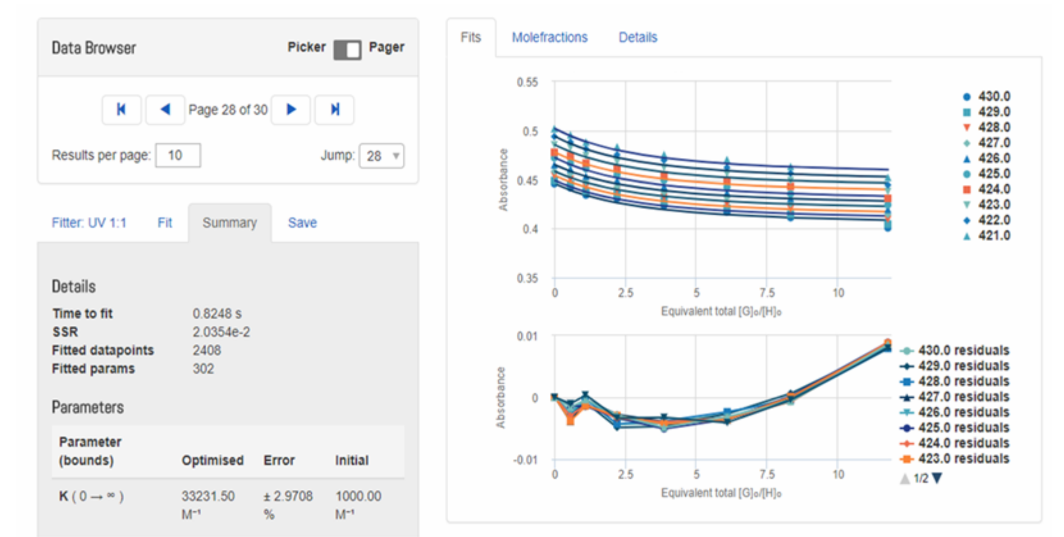

1:1 Uv Binding model for OPC4A **5** with TEAB4

Figure S5: *Top: (left and right side)*: UV–vis spectra of OPC4A **5** (1.18×10^{-5} M to 1.14×10^{-5} M) upon addition of $TEABF_4$ (0–12 equivalents) in 9:1,v/v CH_2Cl_2/CH_3OH. *Bottom:* Screenshot from http://app.supramolecular.org/bindfit/ showing 1:1 binding model for OPC4A **5** with $TEABF_4$

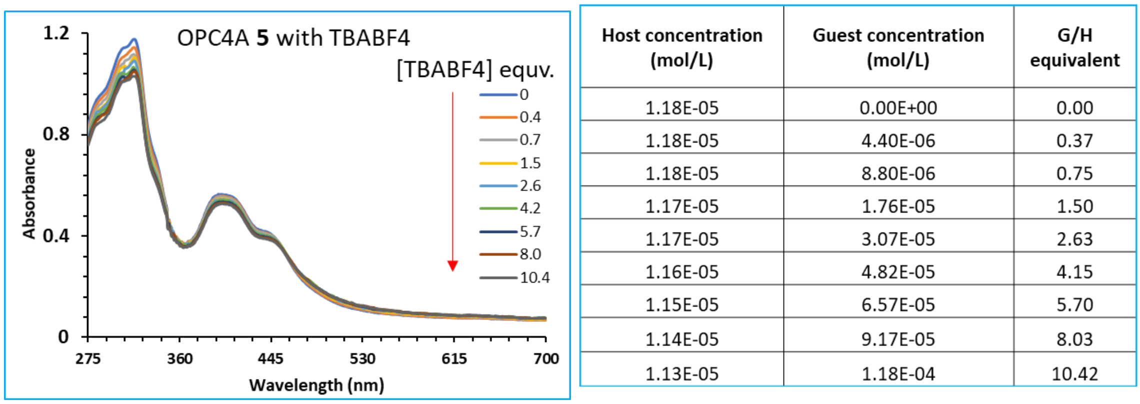

Host concentration (mol/L)	Guest concentration (mol/L)	G/H equivalent
1.18E-05	0.00E+00	0.00
1.18E-05	4.40E-06	0.37
1.18E-05	8.80E-06	0.75
1.17E-05	1.76E-05	1.50
1.17E-05	3.07E-05	2.63
1.16E-05	4.82E-05	4.15
1.15E-05	6.57E-05	5.70
1.14E-05	9.17E-05	8.03
1.13E-05	1.18E-04	10.42

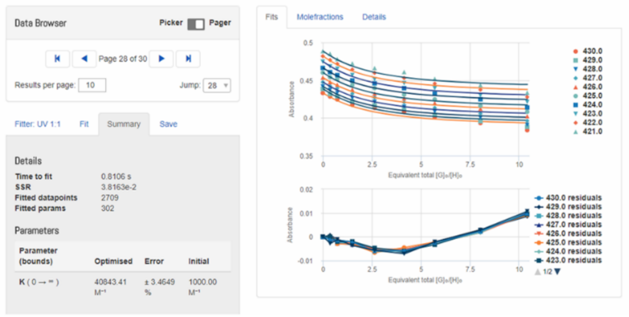

1:1 Uv Binding model for OPC4A **5** with TBABF4

Figure S6: *Top: (left and right side)*: UV–vis spectra of OPC4A **5** (1.18×10^{-5} M to 1.14×10^{-5} M) upon addition of $TBABF_4$ (0–10 equivalents) in 9:1,v/v CH_2Cl_2/CH_3OH. *Bottom:* Screenshot from http://app.supramolecular.org/bindfit/ showing 1:1 binding model for OPC4A **5** with $TBABF_4$.

References

1. (a) P. Thordarson, *Chem. Soc. Rev.*, 2011, **40**, 1305-1323; (b) http://app.supramolecular.org/bindfit/

Table SI1. DFT Calculations

Job Number	Job Name	Route	Stoichiometry	RwB97XD Energy (Hartree)	2600	
55985	OPCA Cone Gen ECP PLUS DCM ex 5253	#N wB97xD/GenECP OPT SCRF=(PCM,Solvent=Dichloromethane) Geom=Check	C92H64	-3543.05966532		
55980	TBACl in DCM wB97xD/GenECP from 2327	#N wB97xD/GenECP OPT SCRF=(PCM,Solvent=Dichloromethane) Geom=Check	C16H36ClN	-700.9470509		
				-4244.006716		
56212	Scott cone plus TBACl in DCM ex 5643	#N wB97xD/GenECP OPT SCRF=(PCM,Solvent=Dichloromethane) Geom=Check	C108H100ClN	-4244.136641		
				-0.129925185	**-337.805**	**kJ/mol**
55985	OPCA Cone Gen ECP PLUS DCM ex 5253	#N wB97xD/GenECP OPT SCRF=(PCM,Solvent=Dichloromethane) Geom=Check	C92H64	-3543.05966532		
55977	TBABr in DCM wB97xD/GenECP from 2327	#N wB97xD/GenECP OPT SCRF=(PCM,Solvent=Dichloromethane) Geom=Check	C16H36BrN	-699.1793985		
				-4242.239064		
56210	Scott cone plus TBABr in DCM	#N wB97xD/GenECP OPT SCRF=(PCM,Solvent=Dichloromethane) Geom=Check	C108H100BrN	-4242.36025		
				-0.12118209	**-315.073**	**kJ/mol**
55985	OPCA Cone Gen ECP PLUS DCM ex 5253	#N wB97xD/GenECP OPT SCRF=(PCM,Solvent=Dichloromethane) Geom=Check	C92H64	-3543.05966532		
56061	TBAI in DCM Gen ECP wB97xD/6-31G(d) from 23	#N wB97xD/GenECP OPT SCRF=(PCM,Solvent=Dichloromethane) Geom=Check	C16H36IN	-697.4036638		
				-4240.463329		
56196	Scott plus TBAI and DCM ex 5484 new	#N wB97xD/GenECP OPT SCRF=(PCM,Solvent=Dichloromethane) Geom=Check	C108H100IN	-4240.585022		
				-0.121692956	**-316.402**	**kJ/mol**
6021	OPCA Cone PLUS DCM ex 5253	#N wB97xD/6-31G(d) OPT SCRF=(PCM,Solvent=Dichloromethane) Geom=Connec	C92H64	-3543.110208		
6027	TMABF4 in DCM wB97xD/6-31G9d) from 2275	#N wB97xD/6-31G(d) OPT SCRF=(PCM,Solvent=Dichloromethane) Geom=Connec	C4H12BF4N	-638.6789257		
				-4181.789133		
6015	SCOTT + TMABF4 + DCM ex 5174	#N wB97xD/6-31G(d) OPT SCRF=(PCM,Solvent=Dichloromethane) Geom=Check	C96H76BF4N	-4181.849108		
				-0.05997502	**-155.935**	**kJ/mol**
6021	OPCA Cone PLUS DCM ex 5253	#N wB97xD/6-31G(d) OPT SCRF=(PCM,Solvent=Dichloromethane) Geom=Connec	C92H64	-3543.110208		
6026	TEABF4 in DCM wB97xD/6-31G(d) from 2298	#N wB97xD/6-31G(d) OPT SCRF=(PCM,Solvent=Dichloromethane) Geom=Connec	C8H20BF4N	-795.8947434		
				-4339.004951		
5994	OPCA cone plus TEABF4 in DCM	#N wB97xD/6-31G(d) OPT SCRF=(PCM,Solvent=Dichloromethane) Geom=Connec	C100H84BF4N	-4339.06834		
				-0.063389111	**-164.812**	**kJ/mol**
6021	OPCA Cone PLUS DCM ex 5253	#N wB97xD/6-31G(d) OPT SCRF=(PCM,Solvent=Dichloromethane) Geom=Connec	C92H64	-3543.110208		
6022	TBABF4 in DCM wB97xD/6-31G(d) from 2359	#N wB97xD/6-31G(d) OPT SCRF=(PCM,Solvent=Dichloromethane) Geom=Connec	C16H36BF4N	-1110.3206		
				-4653.430808		
6014	OPCA plusTBABF4 in DCM EX 5173	#N wB97xD/6-31G(d) OPT SCRF=(PCM,Solvent=Dichloromethane) Geom=Check	C108H100BF4N	-4653.507282		
				-0.07647396	**-198.832**	**kJ/mol**

Note: 1 Hartree = 2600 kJ/mol
